# Monocular Elevation Deficiency: A Case Series of Surgical Outcome

**Published:** 2014-03

**Authors:** Mohammad Reza Talebnejad, Gholam Abbas Roustaei, Mohammad Reza Khalili

**Affiliations:** Poostchi Ophthalmology Research Center, Department of Ophthalmology, Shiraz University of Medical Sciences, Shiraz, Iran

**Keywords:** Monocular elevation deficiency, Strabismus, Surgery, Recession

## Abstract

**Background:** Inferior rectus recession, Knapp procedure, partial tendon transposition, and combined procedure are different surgical procedures in the management of monocular elevation deficiency (MED). Only a few studies have been published on the management of this problem. In this study, we report our experience with patients with MED focusing on the indications and types of surgery in the south of Iran.

**Methods: **In this case series, a computerized database review on 4773 patients with strabismus was performed and 18 patients diagnosed as having MED who had undergone strabismus surgery were enrolled.

**Results:** Of the 18 patients, 13 had only hypotropia and 5 had horizontal deviation as well. Preoperative vertical deviation was between 15 and 60 prism diopter (mean±SD=25.8±10.7 PD). Fourteen patients had positive forced duction test on elevation. Seventeen patients had ptosis twelve of them had true ptosis and the remaining 5 had pseudoptosis). The mean postoperative follow-up was 24.4 months. Four patients underwent Knapp procedure, 12 patients underwent inferior rectus recession, and for 2 patients a combined procedure was performed. The mean postoperative hypotropia was 6.1±7.9 PD. Twelve out of the 18 patients were corrected to within five PD of orthophoria and no one was found with overcorrection.

**Conclusion:** Although MED is etiologically multifactorial, satisfactory surgical results can be achieved by judicious selection of the surgical technique based on the results of the forced duction test.

## Introduction

Monocular elevation deficiency (MED) is classified as three subtypes: 1) restrictive form, with features including positive forced duction test (FDT) for elevation, normal elevation forced generation test (FGT), and elevation saccadic velocity, often an extra or deeper lower eyelid fold on attempted upgaze and poor or absent Bell phenomenon; 2) paretic form with elevator muscle weakness, with features including free FDT, reduced elevation FGT and saccadic velocity, in which the Bell phenomenon is often preserved; and 3) a combination form, with features including positive FDT for elevation and reduced FGT and saccadic velocity for elevation.^1^

Indications for surgery are vertical deviation in primary gaze, deviation-induced amblyopia, diplopia in primary gaze, and restricted binocular fields.^2^ The goal of surgery is to improve the position of the affected eye in primary gaze, by increasing the field of binocular vision. If restriction to upgaze is demonstrated on the FDT, inferior rectus muscle (IR) restriction is present. An IR recession (IRR) with conjunctival recession should be done in such patients. In cases of secondary IR restriction, the hypotropia will persist after IRR because of primary superior rectus muscle (SR) palsy. In such cases, a Knapp procedure should be performed in addition to IRR.^2^ If the FDT is non-restrictive, the affected patient has either SR paresis or supranuclear MED and the Knapp procedure should be performed.^3^ A partial tendon transposition could be considered if a patient has a prior IRR, and has <25 prism diopter (PD) vertical deviation in primary gaze, or if the patient does not have a prior IRR and the deviation in primary gaze is <10 PD.^4^ In the Knapp procedure, all the tendons of the medial and lateral rectus muscles are transposed to the insertion of the superior rectus muscle, whereas in the partial Knapp procedure, half of the tendons of the medial and lateral rectus muscles are transposed to the insertion of the superior rectus muscle.^1^

The purpose of this case series was to evaluate the results of different surgical procedures based on the results of the FDT in patients with MED in our center. It is the first report of different surgical procedures in patients with MED in the south of Iran. 

## Patients and Methods

In this case series, a computerized database review was performed at our tertiary ophthalmology center on 4773 patients with strabismus who had undergone strabismus operation between August 2006 and May 2012, searching for patients with MED. A case series retrospective chart review was performed and patients with a positive history of trauma or with a diagnosis of myasthenia gravis, thyroid ophthalmopathy, and Brown syndrome were excluded. Finally, 18 patients diagnosed as having MED who had undergone strabismus surgery were enrolled. The study was registered with our institutional Review Board and approved by the institutional Ethics Committee. 

Complete ophthalmic examination, visual acuity assessment, ocular motility, slit lamp examination, external eye examination, indirect ophthalmoscopy, and refraction were performed. Visual acuity assessment was according to the standard Snellen chart in cooperative patients and fixation pattern in preverbal children. Pre- and postoperative eye deviation measurements were based on the prism-cover test in adults and the Hirschberg test on children without cooperation. The evaluation of the FDT was done at the operating room before surgery, and surgical planning was based on the obtained results. The upgaze limitation of the patients was assessed clinically, and the results were graded from -1 to -4, as follows: mild limitation=-1; moderate limitation=-2; severe limitation=-3; and no elevation above primary position=-4. All the surgeries were done or supervised by the first author.

The Wilcoxon Signed Ranks test was used to compare the preoperative and postoperative values, and the Kruskal Wallis Test was used to assess intergroup differences. P<0.05 was considered statistically significant.

## Results

Eighteen patients diagnosed as having MED who had undergone strabismus surgery in our department were enrolled. Thirteen patients were men and 5 were women. The patients were 3 to 53 years old (mean: 15.5±11.8 years). Nine patients had right eye and nine had left eye involvement. Thirteen patients had only vertical deviation, and the remaining 5 patients had vertical and horizontal deviation. Preoperative vertical deviation was between 15 and 60 PD (mean±SD=25.8±10.7 PD). Preoperative horizontal deviation was between 15 and 25 PD exodeviation in 4 patients and 20 PD esodeviation in one patient. Fourteen patients had positive FDT on elevation. Twelve patients had true ptosis and 5 had pseudoptosis. In only one patient ptosis was not present. One patient had true ptosis with the Marcus-Gunn jaw winking phenomena. 

The mean postoperative follow-up period was 24.4±21.5 months (range: 1-60 months). Four patients underwent the Knapp procedure, and one patient underwent partial tendon Knapp procedure combined with horizontal muscle recession (table 1). Twelve patients underwent IRR and 2 patients underwent IRR combined with horizontal recession (table 2). The average correction of hypotropia was 18.6 PD from an average preoperative deviation of 25.4 PD (P=0.002). One patient underwent IRR combined with the Knapp procedure at the same session and one patient with prior IRR underwent partial tendon Knapp procedure 4 months later (table 3). Preoperative limitation of upgaze was -2 to -4 (mean: -3.5) and postoperatively it was -1 to -3 (mean: -1.55). This finding indicated a significant decrease in upgaze limitation (P<0.001, tables 1,2 and 3).

**Table 1:** Surgical results of the patients undergoing the Knapp procedure

**No**	**FDT**	**Limit of upgaze**		**Eye deviation (PD)**		**Amount of correction (PD)**	**Operation**	**F/U**	**Ptosis**
		**Preop**	**Postop**	**Preop**	**Postop**				
1	-	-3	-1	30 RHoT 15 XT	15 XT	30	Knapp	9	True
2	-	-3	-2	30 LHoT	1o LHoT	20	Knapp	58	True+MGJW
3	-	-3	-1	20 LHoT	5 LHoT	15	Knapp	60	No
4	-	-2	-1	15 RHoT 25 XT	Ortho	15	Partial tendon Knapp+RLRR	8	Pseudo

FDT: Forced duction test; F/U: Follow-up; Knapp: Knapp procedure; LHoT: Left hypotropia; Limit: Limitation; MGJW: Marcus Gunn jaw winking; Ortho: Orthophoria; PD: Prism diopter; Pseudo: Pseudoptosis; RHoT: Right hypotropia; RLRR: Right lateral rectus recession; XT: Exotropia

**Table 2:** Surgical results of the patients undergoing IRR

**No**	**FDT**	**Limit of upgaze**		**Eye deviation (PD)**		**Amount of correction (PD)**	**Operation**	**F/U**	**Ptosis**
		**Preop**	**Postop**	**Preop**	**Postop**				
5	+	-4	-2	30 LHoT	5 LHoT	25	LIRR	8	True
6	+	-3	-2	25 LHoT	10 LHoT	15	LIRR	13	True
7	+	-4	-2	20 RHoT	10 RHoT	10	RIRR	6	True
8	+	-4	-1	30 RHoT	5 RHoT	25	RIRR	40	True
9	+	-4	-1	25 LHoT 15 XT	15 XT	25	LIRR	47	Pseudo
10	+	-3	-1	15 RHoT	Ortho	15	RIRR	20	Pseudo
11	+	-4	-2	25 LHoT 20 ET	5 LHoT	20	LIRR+LMR recess	30	Pseudo
12	+	-3	-1	15 RHoT	Ortho	15	RIRR	10	True
13	+	-3	-1	15 RHoT	Ortho	15	RIRR	1	True
14	+	-4	-3	60 LHoT	30 LHoT	30	LIRR^*^	3	True
15	+	-4	-2	25 LHoT	12 LHoT	13	LIRR	55	True
16	+	-4	-1	20 RHoT 20 XT	5 RHoT 10 XT	15	RIRR+RLRR	3	True

ET: Esotropia; FDT: Forced duction test; F/U: Follow-up; LHoT: Left hypotropia; Limit: Limitation; LIRR: Left inferior rectus recession; LLRR: Left lateral rectus recession; Ortho: Orthophoria; PD: Prism diopter; Pseudo: Pseudoptosis; RHoT: Right hypotropia; RIRR: Right inferior rectus recession; RLRR: Right lateral rectus recession; XT: Exotropia; *This patient has been scheduled for subsequent surgery (partial tendon Knapp).

**Table 3:** Surgical results of the patients undergoing combined procedure

**No**	**FDT**	**Limit of upgaze**		**Eye deviation (PD)**		**Amount of correction (PD)**	**Operation**	**F/U**	**Ptosis**
		**Preop**	**Postop**	**Preop**	**Postop**				
17	+	-4	-3	30 LHoT	8 LHoT	22	LIRR recess+(Knapp)^*^	52	Pseudo
18	+	-4	-1	35 RHoT	5 RHoT	30	RIRR+Knapp	16	True

FDT: Forced duction test; F/U: Follow-up; Knapp: Knapp procedure; LHoT: Left hypotropia; Limit: Llimitation; PD: Prism diopter; Pseudo: Pseudoptosis; RHoT: Right hypotropia; RIRR: Right inferior rectus recession; *This patient underwent partial tendon Knapp procedure 4 months after IR recession with eight PD hypotropia 48 months after the second operation

The mean postoperative vertical deviation was 6.11±7.9 PD. Compared to preoperative measurements, there was a mean correction of 19.7 PD in the amount of hypotropia in primary gaze position. 

## Discussion

In this case series, we performed different surgical procedures based on the results of the FDT in patients with MED and evaluated the results based on ocular alignment in primary position. 

The pathophysiology of MED is poorly understood. The early description of this condition was thought to be due to a combination of SR and inferior oblique muscle palsy (called double elevator palsy). Studies have shown that only 30% of cases are caused by this problem, and the FDT has demonstrated that 70% is caused by IR restriction.^5^ Magnetic resonance imaging (MRI) may be a useful adjunct to saccadic velocity assessment in differentiating between primary IR restriction, primary SR paresis, and congenital supranuclear elevation deficiency.^6^

In our study, MED had similar predilection for the right eye and left eye involvement: 9 patients had right eye and 9 had left eye involvement. A predilection to right side involvement has been reported in MED in the series reported by Ziffer et al.^7^ and Kucak and co-workers.^8^ On the other hand, Khawam and Younis^9^ and also Bagheri et al.^10^ reported more instances of left eye involvement. Considering the mentioned studies and ours, it seems that the laterality of the condition provides no particular diagnostic information.

The goal of surgery in MED associated with ptosis or pseudoptosis is the management of combined hypotropia and blepharoptosis. For surgical correction of MED, the procedure of choice is determined by the FDT, which ascertains whether the cause is paretic or restrictive. In the presence of SR palsy (paretic form), the procedure employed is a Knapp transposition. The transposition procedure is not recommended in the presence of IR restriction. Therefore, it is important to perform FDT prior to surgery. In our series, the mean amount of correction with the Knapp procedure alone was 20.0 PD. In his original work, Knapp^3^ reported 15 patients with MED and good results were obtained in 14 out of the 18 patients (93%). Correction of hypotropia in his study ranged from 21 to 55 PD with a mean of 38 PD.

Others have found similar amounts of correction. Barsoum-Homsy^11^ observed an average correction of 31.7 PD and Watson^12^ in his series observed a mean correction of 30.5 PD after the Knapp procedure. Cooper and Greenspan^13^ reported 26.6 PD correction of hypotropia after this procedure. Scott^14^ performed the Knapp procedure in 19 patients and observed 21.1 PD corrections. Bandyopadhyay et al.^15^ reported a correction of 29.4 PD of vertical deviation. Kalmesh and Dadeya^16^ in their series of MED with associated horizontal deviation noted a correction of 20 PD of horizontal and 25 PD of vertical deviation. In our series, 4 patients underwent the Knapp procedure and one patient underwent partial tendon Knapp procedure combined with horizontal muscle recession. We observed a mean correction of 20.0 PD with the Knapp procedure, a finding similar to most of the mentioned studies.^3^^,^^4^^,^^8^^,^^10^^-^^13^

Most patients with MED have IR restriction according to a large number of studies. In our study, 14 patients had positive FDT on elevation; IR restriction was present in 14 out of the 18 patients (77.7 %). This high percentage of IR restriction in patients with MED has been reported by other authors.^14^^,^^15^^,^^17^ An IRR should be done in such patients. In our study, 12 patients underwent only IR recession for the management of MED. The average correction was 18.6 PD from an average preoperative deviation of 25.4 PD. There are a few reports on the results of only IR recession for the management of MED. In the study performed by Bandyopadhyay and colleagues,^15^ the average correction for IR recession was 16 PD from an average preoperative deviation of 25.8 PD. Kocak-Altintas AG et al.^18^ reported an average correction of 12.27 after IR recession from an average preoperative deviation of 29.17 PD. In another report by Kocak-Altintas AG and co-workers,^8^ vertical deviation was adequately corrected after IR recession in only one patient; the other 5 patients then underwent transposition surgery 6 months later.^8^ In a study performed by Bagheri and colleagues,^10^ one patient with 30 PD hypotropia underwent IR recession alone because of severe restriction on the FDT; the amount of correction was 20 PD.

If hypotropia persists after IRR, in the presence of the residual SR palsy, IRR needs to be followed by the Knapp procedure. In our series, one patient with prior IRR underwent partial tendon Knapp procedure 4 months later. In this patient with 30 PD hypotropia, after IRR, there was 20 PD residual hypotropia. Because of residual SR palsy, partial tendon Knapp procedure was performed 4 months later. After the second procedure, the amount of hypotropia was 8 PD. In another patient because of the high amount of hypotropia (35 PD) and moderately positive FDT, we decided to perform a combined procedure at the same session. The amount of residual hypotropia in this patient was 5 PD. The average correction of hypotropia with the combined procedure in these two patients was 26 PD. In the series of 28 patients with MED reported by Bandyopadhyay et al.^15^ three patients underwent combined surgeries, with an average correction of 28.6 PD of deviation at the end of two surgeries.

Kocak-Altimtas and colleagues,^8^ reported a series of 6 patients with MED and positive FDT who underwent IRR, followed by the Knapp procedure. A mean correction of 25.8±5.6 PD was achieved after the combined procedure. Scott^14^ reported an average correction of 38 PD following a combined procedure. An average correction of 23.75 PD was achieved after simultaneous Knapp and IRR in the series reported by Bagheri et al.^10^ Burke^4^ found a statistically significant difference in the magnitude of vertical correction in patients with an IRR performed prior to the Knapp surgery (38 PD) compared with those with no prior IRR (21 PD).

According to our results, the mean residual deviation was 3.8 PD after Knapp, 6.8 PD after IRR, and 6.5 PD after combined procedure. This finding may be attributable to the larger magnitude of preoperative vertical deviation in patients who underwent a combined procedure. 

In our series, out of the 18 patients with MED, 12 (66.7%) patients were corrected to within 5 PD of orthophoria, 16 (88.9%) patients within 10 PD of orthophoria, and no one was found with overcorrection. In a series of 28 patients with MED reported by Bandyopadhyay et al.^15^twenty-four out of 28 patients (86%) had correction of deviation to within 10 PD, a finding similar to our results. Overall preoperative mean vertical deviation was 25.8±10.7 PD and postoperative deviation was 6.11±7.9 PD with an average 19.7 PD correction of hypotropia.

One limitation of our study is that we did not perform sensory neural tests such as the stereopsis test. Although not an objective of our study, this test might have added some information regarding the sensory results of the procedures. This could be assessed in future studies.

## Conclusion

Although MED is etiologically multifactorial, satisfactory surgical results can be achieved by judicious selection of the surgical technique based on the results of the FDT. If restriction to upgaze is demonstrated on the FDT, IRR could be done. In cases of secondary IR restriction, hypotropia will persist after IRR because of primary SR palsy. In such cases, a Knapp procedure should be performed in addition to IRR. If the result of the FDT is negative, the patient has either SR paresis or supranuclear MED and the Knapp procedure should be performed. 
